# Palaearctic species of Rhamphomyia (Pararhamphomyia) anfractuosa group (Diptera, Empididae)

**DOI:** 10.3897/zookeys.514.9379

**Published:** 2015-07-27

**Authors:** Miroslav Barták, Štěpán Kubík

**Affiliations:** 1 Department of Zoology and Fisheries, Faculty of Agrobiology, Food and Natural Resources, Czech University of Life Sciences Prague, CZ-16521 Praha 6-Suchdol, Czech Republic

**Keywords:** Empidoidea, *Rhamphomyia*, taxonomy, key, new species, Palaearctic

## Abstract

Palaearctic species of the Rhamphomyia (Pararhamphomyia) anfractuosa group are revised. Rhamphomyia (Pararhamphomyia) biflexata **sp. n.**, Rhamphomyia (Pararhamphomyia) lineodorsata **sp. n.**, Rhamphomyia (Pararhamphomyia) nudiscutellata **sp. n.**, and Rhamphomyia (Pararhamphomyia) shatalkini **sp. n.** (all from Russian Far East) are described and illustrated. A key to Palaearctic species of the Rhamphomyia (Pararhamphomyia) anfractuosa group is provided.

## Introduction

*Rhamphomyia* Meigen is one of the three megadiverse groups of Empididae, alongside *Empis* Linnaeus and *Hilara* Meigen. Almost 600 species, distributed mostly in the Northern Hemisphere have been described worldwide (Yang et al. 2007; Barták 2007; Barták et al. 2007; Barták and Kubík 2008a, b, c, 2009, 2010, 2012; Saigusa 2012; Barták et al. 2014), but many more await description.

**Figures 1–4.**
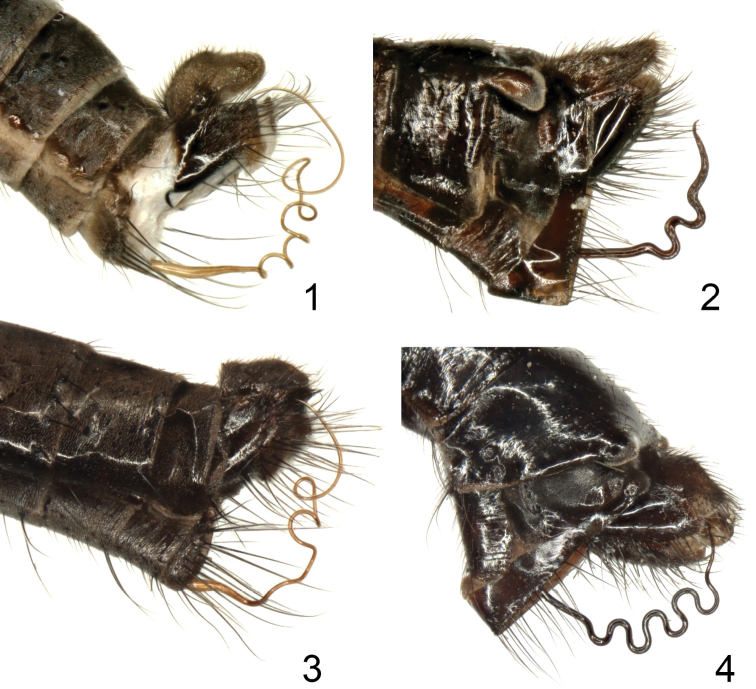
Male terminalia, lateral view: **1** Rhamphomyia (Pararhamphomyia) anfractuosa Bezzi **2** Rhamphomyia (Pararhamphomyia) biflexata sp. n. **3** Rhamphomyia (Pararhamphomyia) lineodorsata sp. n. **4** Rhamphomyia (Pararhamphomyia) multisinuosa Frey.

Rhamphomyia (Pararhamphomyia) anfractuosa group is delimited here as probably a natural group of *Pararhamphomyia* (as fixed by Barták and Sinclair 2003 and diagnosed by Barták 1982). The most important characters shared by members of this group are as follows (* for characters considered synapomorphic):

- acrostichals present, biserial
- male head holoptic
- legs brown to black
- proepisternal depression without setae
- phallus forms several tightly coiled loops*
- cercus simple (“subcercal process” or “posterior cercus” absent)*
- hypandrium membranose posteriorly, anterior part desclerotized medially*
- tip of epandrial lamellae with expanding membranose pouch (see Fig. 1 and 9)*
- tergite and sternite 8 fused*
- axillary angle acute to right angled
- anal vein complete or depigmented in middle
- halter yellow
- female leg parts broadly pennate

As usual in *Rhamphomyia*, it is not always easy to arrange single females into groups or even subgenera (compare species group approach in Barták 2002, 2003; Barták and Kubík 2009), so, separate key to females of this group would be meaningless.

The most allied species to this group are species of *Pararhamphomyia* sharing most characters with this group mentioned above, except the tightly coiled phallus and/or yellow halter (e.g., *Rhamphomyia slovaki* Barták, *Rhamphomyia plumifera* Zetterstedt, *Rhamphomyia deformata* Frey, *Rhamphomyia deformatella* Barták). The subgenus *Calorhamphomyia* Saigusa with similarly modified last abdominal segments and phallus may be allied to this group of species, however its members may be easily separated by at least partly yellow legs.

## Material and methods

The material studied is deposited in the following collections: CULSP (Czech University of Life Sciences Prague), ZMMU (Zoological Museum of Moscow State University). Acronyms are used further in the text.

**Figures 5–8.**
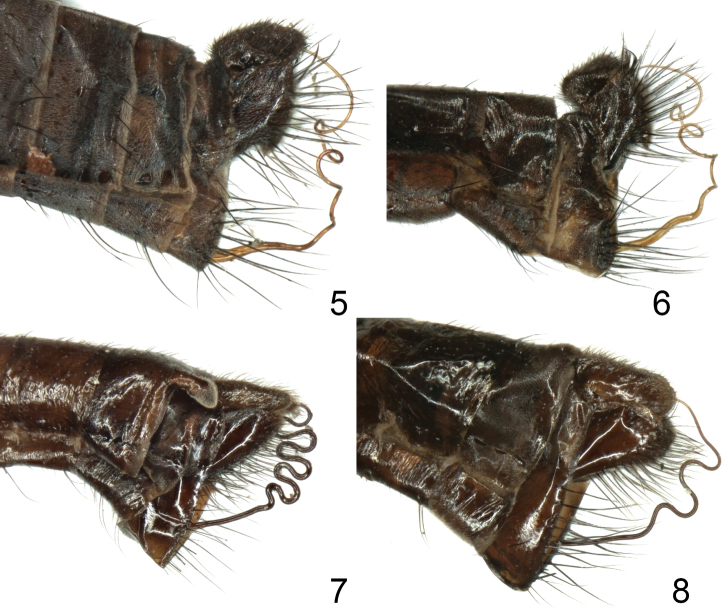
Male terminalia, lateral view: **5** Rhamphomyia (Pararhamphomyia) nudiscutellata sp. n. **6** Rhamphomyia (Pararhamphomyia) robustior Frey **7** Rhamphomyia (Pararhamphomyia) shatalkini sp. n. **8** Rhamphomyia (Pararhamphomyia) sp. 2.

**Figures 9–12.**
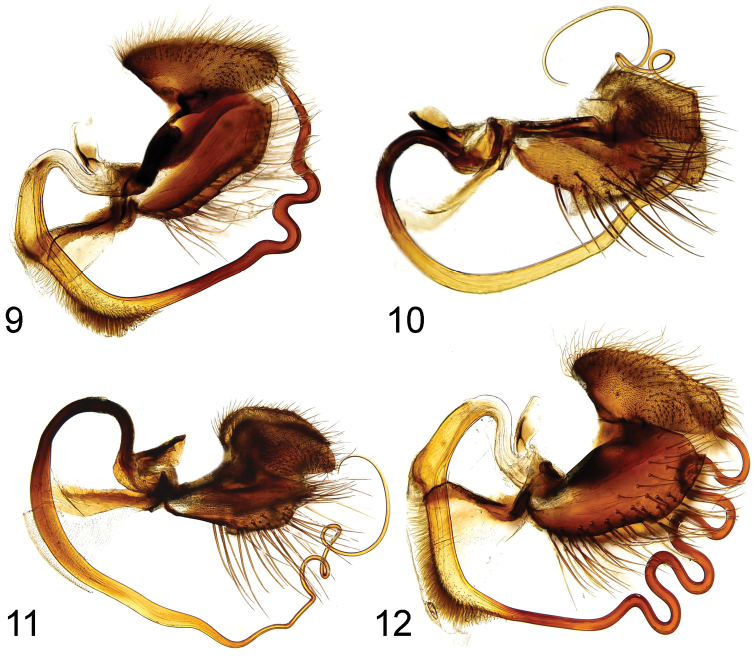
Male genitalia (macerated), lateral view: **9** Rhamphomyia (Pararhamphomyia) biflexata sp. n. **10** Rhamphomyia (Pararhamphomyia) lineodorsata sp. n. **11** Rhamphomyia (Pararhamphomyia) nudiscutellata sp. n. **12** Rhamphomyia (Pararhamphomyia) shatalkini sp. n.

**Figures 13–16.**
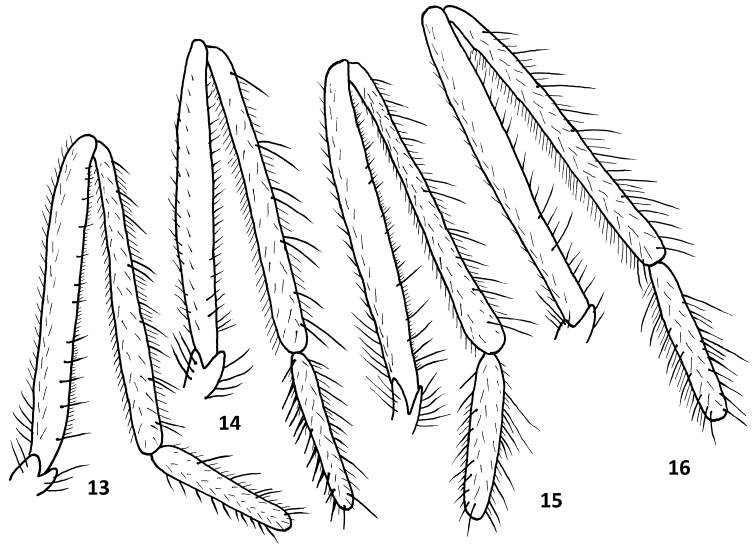
Male hind legs (femur, tibia and basitarsus): **13** Rhamphomyia (Pararhamphomyia) lineodorsata sp. n. **14** Rhamphomyia (Pararhamphomyia) biflexata sp. n. **15** Rhamphomyia (Pararhamphomyia) nudiscutellata sp. n. **16** Rhamphomyia (Pararhamphomyia) shatalkini sp. n.

Terminal abdominal segments (Figs 1–8) and hind legs (Figs 13–16) were photographed by means of Nikon Digital sight DS Fi-1. Each image resulted usually from combining 7–10 layers by means of Nish element. Legs were illustrated from these images; details were added by direct observation. Genitalia (Figs 9–12) together with 2–3 pregenital segments were removed and macerated in potassium hydroxide solution (approx. 10 %) in small vials submerged in hot water for 1–2 hours. After neutralizing with 8 % acetic acid, the genitalia were dissected in glycerine and their parts photographed by means of an Olympus E-41 digital camera mounted on an Olympus BX51 compound microscope. Images were edited with the computer software Quick Foto micro 2.3 provided with Deep focus 3.1. Each image resulted usually from combining 7–15 layers. Images were improved by means of Adobe Photoshop.

The morphological terms used here follow Merz and Haenni (2000), Sinclair (2000) and Sinclair and Cumming (2006). All body measurements (including body and setae length) were taken from dry specimens (therefore the actual length may differ) by means of an ocular micrometer on a Nikon SMZ 1500 binocular microscope.

Length of antennal segments = length of scape: pedicel: postpedicel: style (in 0.01 mm). Male body length was measured from antennal base to the tip of last abdominal segment (without the genitalia) and female body length from the base of antennae to the tip of the cerci. Wing measurements: M_2_/d = length of vein M_2_: greatest length of discal medial cell (discal cell); CuA_1_ ratio = length of apical: preapical sections of vein CuA_1_; lw/ww = greatest length of wing (from basicosta to apex): greatest width of wing. Length of frons is measured from front margin of front ocellus to antennal base.

## Taxonomic account

### Rhamphomyia (Pararhamphomyia) biflexata sp. n.

Taxon classificationAnimaliaDipteraEmpididae

http://zoobank.org/2715DE4F-1E0A-4636-825F-751DC030B876

Figs 2 , 9 , 14

#### Type material.

HOLOTYPE male, Russia, Amurskaja oblast, g. (= city) Zeja, 8.vii.1981, A. Shatalkin, deposited in ZMMU; paratype: 1 male, same data as holotype (CULSP).

#### Diagnosis.

Species of the subgenus *Pararhamphomyia* with pair of long thumb-like dorsomarginal processes on tergite 6, lustrous abdomen, uniserial dorsocentrals and two outgoing loops of the phallus.

#### Etymology.

The species epithet stresses two outgoing loops on the phallus.

#### Description.

**Male.** Head holoptic, facets in dorsal third of eye enlarged. Frons blackish brown, light grey microtrichose, without setae. Ocellar setae less than half as long as frons, black, ocellar triangle without additional setae. Face blackish brown, light grey microtrichose dorsally and broadly lustrous in ventral third, 0.28 mm broad ventrally and 0.32 mm long, bare. Occiput blackish brown, microtrichose, sparsely black setose, setae rather thick and short, ventrally longer and finer. Antenna black, basal segment brown, length of antennal segments = 11: 12: 40: 19, setae on basal two segments up to 0.10 mm long. Labrum brown, lustrous, slightly longer than head is high. Palpus brown, short, covered with several short setae and one strong preapical seta (0.25 mm long). Gena narrow and lustrous, clypeus lustrous.

Thorax brownish-black, light grey microtrichose (without brownish-yellow tinge), darker stripes below rows of acrostichals and dorsocentrals scarcely visible. All setae black. Chaetotaxy: proepisternum with about 10 setae, both propleural depression and prosternum bare; acrostichals damaged but probably irregularly biserial and short; 8 uniserial dorsocentrals, 0.20 mm long in middle of rows, ending in 2 prescutellars, 1 short intrahumeral, 1 strong posthumeral; postpronotum with 1 strong seta and about 8–10 smaller setae; 3–4 notopleurals (1–2 setae on anterior part of notopleuron); 1 supraalar and 1 prealar; 1 long and 1 shorter postalar; 3 pairs of scutellars (outer pair short); laterotergite with black setae.

Legs brown, lustrous, black setose. Coxae blackish-brown, microtrichose (only hind coxa with lustrous spot anteriorly near base), black setose. One long seta in posteroapical comb of hind tibia. Fore femur with fine anteroventral setae 1/3 as long as long as depth of femur, dorsal setae shorter, posteroventrals almost absent. Fore tibia with 4 posterodorsal setae slightly longer than width of tibia, ventral and anterodorsal setae short. Mid femur with two rows of spiny setae ventrally, anteroventrals half as long as depth of femur, posteroventrals longer than depth of femur, other setae short. Mid tibia with two rows of setae dorsally nearly 2× longer than width of tibia (each row consists of 3–4 setae including preapicals), two rows of ventral setae about as long as width of tibia. Hind femur (Fig. 14) with row of fine anteroventrals shorter than depth of femur, posteroventrals equally fine and irregularly arranged. Hind tibia with 5–6 pairs of antero- and posterodorsal setae 1.5× longer than width of tibia, ventral setae short. Basal tarsomeres of fore and mid legs thin and short setose, with several short ventral spines. Basal tarsomere of hind leg thin, with several setae dorsally 2× longer than diameter of tarsomere and with several ventral spines slightly longer than diameter of tarsomere.

Wing clear to light brownish, stigma slightly darker, veins brown, anal vein almost complete. Costal seta strong and long (several other setae between costal seta and wing base relatively strong and long), axillary angle acute. Measurements: M_2_/d = 1.3, CuA_1_ ratio = 1.6–1.7, lw/ww = 2.5–3.1. Halter yellow, calypter yellow with dark fringes.

Abdomen brown, lustrous (only segment 1 and part of segment 7 microtrichose as well as tips of thumb-like processes). All setae dark. Hind marginal setae on sides of tergites 2–4 nearly as long as their corresponding segments (discal setae shorter), on segments 5–6 shorter (but still slightly longer than discal setae), segment 7 very short setose. Dorsum of abdomen with short setae. Tergite 6 (Fig. 2) with two thumb-like processes on dorsal hind side; tergite 8 fused with sternite. Phallus (Fig. 9) with two outgoing loops; hypandrium membranose on posterior part (this part covers only part of ventral “ciliation” of phallus); phallus with hair-like “ciliation” ventrobasally and not much produced basal bulge.

**Length**: Body 5.5 mm, wing 5.5 mm.

**Female.** Unknown.

#### Remarks.

Rhamphomyia (Pararhamphomyia) biflexata sp. n. may be easily identified according to the key. It is the only species with long dorsomarginal processes on tergite 6 (similarly as *Rhamphomyia shatalkini*) and simultaneously with two outgoing loops on phallus. Female is unknown with certainty, see also Remarks under *Rhamphomyia shatalkini* sp. n.

#### Distribution.

Russia (Far East).

#### Dates of occurrence.

July.

### Rhamphomyia (Pararhamphomyia) lineodorsata sp. n.

Taxon classificationAnimaliaDipteraEmpididae

http://zoobank.org/945770C7-B15A-40B5-B524-5B6548EEC453

Figs 3 , 10 , 13

#### Type material.

Holotype male: Russia, Amurskaja oblast, g. (= city) Zeja, 31.viii.1982, A. Shatalkin, deposited in ZMMU; Paratypes: 2 males, same data as holotype; 1 male, same locality as holotype, 29.viii.1981, A. Shatalkin; paratypes depositories: ZMMU, CULSP.

#### Additional material examined

(excluded from type series): 2 females, same locality as holotype, 4.ix.1981; 1 female, same locality as holotype, 13.ix.1981 – all A. Shatalkin (ZMMU and CULSP).

#### Diagnosis.

Species of the subgenus *Pararhamphomyia* with phallus forming loops in space, dark brownish black mesoscutum with darker stripes below rows of setae and uniserial dorsocentrals. Female tergites 6 and 7 lustrous and hind tibia without pennate ciliation dorsally.

#### Etymology.

The name of the species is derived from dark stripes on the mesoscutum, differing from closely allied species, *Rhamphomyia robustior* Frey.

#### Description.

**Male.** Head holoptic, facets in dorsal half of eye enlarged. Frons blackish brown, light grey microtrichose, without setae. Ocellar setae one third as long as frons, black, ocellar triangle with 1–2 pairs of additional setae. Face blackish brown, light grey microtrichose, 0.30 mm broad ventrally and equally long, without setae. Occiput blackish brown, microtrichose, sparsely black setose, setae rather thick and short, longer and finer ventrally. Antenna black, scape and pedicel brown, length of antennal segments = 16–18: 12: 48–50: 16, setae on basal two segments up to 0.12 mm long. Labrum brown, lustrous, slightly longer than height of head. Palpus brown, short, covered with several setae and one strong preapical seta (0.20 mm long). Gena narrow and lustrous, clypeus microtrichose.

Thorax brownish-black, rather dark brownish grey microtrichose, scutum with distinct darker stripes below rows of acrostichals and dorsocentrals (best visible in posterior view). All setae black. Chaetotaxy: proepisternum with about 10 setae, both propleural depression and prosternum bare; 10–16 narrowly biserial (anteriorly almost uniserial), fine acrostichals about 0.20 mm long; 8–11 almost regularly uniserial dorsocentrals slightly longer (about 0.30 mm in middle of rows), ending in 2–3 long prescutellars, 1–2 strong intrahumerals, 1 strong posthumeral; postpronotum with 2–3 long and 10–15 gradually shorter setae; 4 notopleurals (2–3 long setae on anterior part of notopleuron); 1 long supraalar and 2–3 smaller prealar; 1 long and 1 shorter postalar; 3 pairs of scutellars; laterotergite with black setae.

Legs brown, microtrichose, black setose. Coxae blackish-brown, microtrichose (only hind coxa with lustrous spot anteriorly near base), black setose. One long seta present in comb at tip of hind tibia. Fore femur with fine anteroventral setae up to half as long as depth of femur, dorsal setae shorter, posteroventrals up to half as long as depth of femur, present mostly only on proximal half. Fore tibia with 4–6 posterodorsal setae about as long as width of tibia, ventral and anterodorsal setae short. Mid femur with two rows of spiny setae ventrally, anteroventrals one third as long as depth of femur, posteroventrals slightly longer than depth of femur, other setation short. Mid tibia with only two anterodorsal setae (one subbasal and one preapical – but holotype on one leg atypically with three such setae), and 4–6 posterodorsals slightly longer than depth of tibia, two rows of ventral setae somewhat shorter than width of tibia (several posteroventrals may be longer than remaining ones). Hind femur (Fig. 13) with ventral microtrichosity up to 0.04 mm long, with rather fine anteroventral setae about as long as depth of femur in basal half and sometimes apically, very short to absent on third quarter of femur, posteroventrals present only on extreme base of femur. Hind tibia with 3–5 antero- and 6–8 posterodorsal setae slightly longer than width of tibia, ventral setae short. Basal tarsomeres of fore and mid legs thin and short setose, mid one with several short ventral spines, basal tarsomere of hind leg slightly swollen, with several setae dorsally and spine like setae ventrally up to 2× longer than diameter of tarsomere.

Wing light brown, stigma slightly darker, veins brown (yellowish in basal part of wing), anal vein almost complete or indistinct about middle. Costal seta present, axillary angle acute. Measurements: M_2_/d = 1.4–1.6, CuA_1_ ratio = 1.4–2.1, lw/ww = 2.7–3.1. Halter yellow, calypter brownish-yellow with dark fringes.

Abdomen brown, dark brown microtrichose (dark brown in both lateral and dorsal views), setae all dark. Hind marginal setae almost as long as corresponding segments, discal setae shorter. Dorsum of abdomen with short setae. Phallus (Figs 3, 10) with three twists in space.

**Length**: Body 4.5–5.3 mm, wing 5.1–5.8 mm.

**Female.** Head dichoptic, frons approximately 0.35 mm long and 0.25 mm wide, subparallel, with two rows of 5–7 relatively long setae on sides. Face approximately 0.25 mm long and subequally wide in middle (slightly broadening ventrally), without setae. Palpus lighter than in male, yellowish red. Thorax as in male, only setae shorter. Fore femur with anterodorsal row of almost pennate setae slightly shorter than depth of femur, with anteroventral row of thin setae as long as depth of femur and with posteroventral row of pennate setae slightly longer than depth of femur. Fore tibia as in male, only posterodorsal setae less differentiated. Mid femur with both (antero)dorsal and (postero)ventral pennation about as long as depth of femur. Mid tibia with short subpennate ciliation both dorsally and ventrally in addition to several slightly longer setae on both sides. Hind femur with long pennate ciliation ventrally in addition to several setae and shorter dorsal subpennate ciliation. Hind tibia with two rows of dorsal setae slightly longer than width of tibia, ventral setae short and slightly subpennate. Wing light brown as in male, measurements: M_2_/d = 1.4–1.6, CuA_1_ ratio = 1.5–1.9, lw/ww = 2.8–3.0. Abdomen microtrichose, with tergites 6 to 8 and sternite 8 lustrous and sternite 7 sublustrous. Hind marginal setae on segments 2–6 2/3 as long as corresponding segments.

**Length**: Body 5.3–5.8 mm, wing 5.5–6.0 mm.

#### Remarks.

Rhamphomyia (Pararhamphomyia) lineodorsata sp. n. is closely allied to remaining three Palaearctic species of *Rhamphomyia anfractuosa* group of species with phallus twisted in space, viz *Rhamphomyia anfractuosa* Bezzi, *Rhamphomyia nudiscutellata* sp. n. and *Rhamphomyia robustior*. Most specimens of this complex examined differ from specimens of *Rhamphomyia multisinuosa* complex (with phallus twisted in a single plain) by absence of submedian anterodorsal setae on mid tibia in addition to characters given in the key. However, as mentioned above, holotype of the above described species has atypically one such seta present on one leg. We excluded females from type series because of problems with exact identification of females in this group of species and because we had no pairs taken in copula. But we believe we identified them properly. See under *Rhamphomyia nudiscutellata* for discussions of the differences between females of this complex.

#### Distribution.

Russia (Far East).

#### Dates of occurrence.

August-September.

### Rhamphomyia (Pararhamphomyia) nudiscutellata sp. n.

Taxon classificationAnimaliaDipteraEmpididae

http://zoobank.org/BB33F445-E025-4126-8C47-007857B89D56

Figs 5 , 11 , 15

#### Type material.

Holotype male: Russia, Amurskaja oblast, g. (gorod = city) Zeja, 14.ix.1981, A. Shatalkin, deposited in ZMMU; Paratypes: 2 males, same data as holotype; 4 males, Tuva, okr. (=region) Baj-Chaaka, Berezovka, 5.ix.1973, V. Kovalev; 2 males, same locality, 3.ix.1973, V. Kovalev; 2 males, same locality, 7.ix.1973, V. Kovalev; 2 males, Tuva, okr. Saryg Sep, listvennicznik na granice lesa (= larch on forest boundary), 28.viii.1973, V. Kovalev; 1 male Chitin, r. (=river) Kuenga, vyche (= above) Chernyshevska, 24.viii.1977, V. Kovalev. Paratypes depositories: ZMMU, CULSP.

#### Additional material examined

(excluded from type series): 2 females, Chitin, r. (reka = river) Kuenga, vyche Chernyshevska, 26.viii.1977, V. Kovalev; 1 female, Tuva, okr. (= region) Baj-Chaaka, Berezovka, 5.ix.1973, V. Kovalev; 1 female, same locality, 2.ix.1973, V. Kovalev; 2 females, Tuva, okr. (= region) Shagonar, Ishtii-Khem, 21.viii.1973, V. Kovalev; 1 female, same locality, 24.viii.1973, V. Kovalev; (ZMMU and CULSP).

#### Diagnosis.

Species of the subgenus *Pararhamphomyia* with phallus forming loops in space, light grey mesoscutum and uniserial dorsocentrals. Female front femur with two rows of ventral setae longer than depth of femur, hind tibia not pennate, wing light brownish and tergites 6 and 7 lustrous.

#### Etymology.

The name of the species is derived from the relatively naked scutellum bearing only four setae (nudus, Latin = naked).

#### Description.

**Male.** Head holoptic, facets in dorsal half of eye enlarged. Frons blackish brown, light grey microtrichose, without setae. Ocellar setae one third as long as frons, black, ocellar triangle with 1–2 pairs of additional setae. Face blackish brown, light grey microtrichose, 0.20 mm broad ventrally and 0.25 mm long, without setae. Occiput brownish black, rather light grey microtrichose, black setose. Antenna black, scape and pedicel brown, length of antennal segments = 15–16: 11: 45–50: 12–14, setae on basal two segments about 0.10 mm long. Labrum brown, lustrous, slightly shorter than head is high. Palpus brown, short, covered with several rather long setae (0.20 mm long), preapical seta poorly differentiated. Gena narrow and lustrous, clypeus microtrichose.

Thorax brownish-black, rather light grey microtrichose, scutum without stripes, only in immature specimens with poorly visible darker stripes below rows of acrostichals and dorsocentrals. All setae black. Chaetotaxy: proepisternum with 5–8 setae, both propleural depression and prosternum bare; 6–10 biserial, short and fine acrostichals (about 0.15 mm long); 7–10 regularly uniserial dorsocentrals (about 0.25 mm in middle of rows), ending in 2 long prescutellars, 1–2 fine and long intrahumerals, 1 strong posthumeral; postpronotum with 2–3 long and 6–10 gradually shorter setae; 3 notopleurals (0–2 long setae on anterior part of notopleuron); 1 long supraalar, prealar absent; 1 long and 1 shorter postalar; 2 pairs of scutellars; laterotergite with black setae.

Legs brown, microtrichose, black setose. Coxae blackish-brown, microtrichose (only hind coxa with lustrous spot anteriorly near base), black setose. One long seta present in comb at tip of hind tibia. Fore femur with complete rows of fine antero- and posteroventral setae up to 2/3 as long as long as depth of femur, dorsal setae shorter. Fore tibia short setose, without differentiated setae except preapical. Mid femur with two rows of spiny setae ventrally, anteroventrals up to one-third as long as depth of femur, posteroventrals slightly longer than depth of femur, other setation short. Mid tibia with only two anterodorsal setae (one short subbasal and one long preapical), and 3–4 posterodorsals slightly longer than depth of tibia, two rows of ventral setae somewhat shorter than width of tibia (several posteroventrals may be longer than remaining ones). Hind femur (Fig. 15) with ventral microtrichosity up to 0.05 mm long, with rather fine anteroventral setae about as long as depth of femur in basal half and in some specimens apically, very short to absent on third quarter of femur, posteroventrals present only on extreme base of femur. Hind tibia with 3–4 antero- and 5–6 posterodorsal setae slightly longer than width of tibia, ventral setae short. Basal tarsomeres of fore and mid legs thin and short setose, mid basal tarsomere with several short ventral spines; basal tarsomere of hind leg slightly swollen, with several setae dorsally and spine like setae ventrally up to 2× longer than diameter of tarsomere.

Wing clear, stigma scarcely darker, veins brown and yellowish in basal part of wing, anal vein indistinct about middle. Costal seta present, axillary angle right. Measurements: M_2_/d = 1.2–1.7, CuA_1_ ratio = 1.6–1.9, lw/ww = 2.7–3.3. Halter yellow, calypter yellow with dark fringes.

Abdomen brown, entirely light grey microtrichose (light grey from both lateral and dorsal views), setae all dark. Hind marginal setae on tergites 2–3 somewhat longer and on tergites 4–6 as long as corresponding segments, discal setae shorter. Dorsum of abdomen with short setae. Phallus (Figs 5, 11) with three twists in space.

**Length**: Body 3.8–4.0 mm, wing 4.5–5.8 mm.

**Female.** Head dichoptic, frons approximately 0.25 mm long and 0.20 mm wide, subparallel, with two rows of 4–6 relatively long setae on sides. Face approximately 0.20 mm long and subequally wide in middle (strongly divergent ventrally), without setae. Palpus brown as in male. Thorax as in male. Fore femur with both antero- and posteroventral rows of setae as long as depth of femur, posteroventrals mostly thin but in some specimens on one or both legs thickened – almost pennate, dorsal ciliation short and thin. Fore tibia as in male, only posteroventral setae slightly differentiated. Mid femur with both (antero)dorsal and (postero)ventral pennation about as long as depth of femur. Mid tibia short setose, most specimens with several antero- and posteroventral setae and/or several setae dorsally shorter than depth of tibia. Hind femur with long pennate ciliation ventrally in addition to several setae and shorter dorsal subpennate ciliation. Hind tibia slightly flattened, with two rows of dorsal setae slightly longer than width of tibia, ventral setae short, short ciliation slightly subpennate. Wing clear with only indistinct brownish tinge, measurements: M_2_/d = 1.3–1.6, CuA_1_ ratio = 1.6–1.8, lw/ww = 2.8–3.0. Abdomen microtrichose, with tergites 6 to 8 and sternites 7 and 8 lustrous. Hind marginal setae on segments 2–4 as long as corresponding segments, on segments 5–7 2/3 as long as corresponding segments.

**Length**: Body 4.0–4.4 mm, wing 4.6–5.2 mm.

#### Remarks.

Rhamphomyia (Pararhamphomyia) nudiscutellata sp. n. is closely allied to the remaining three Palaearctic species of the *Rhamphomyia anfractuosa* group of species with phallus twisted in space, viz *Rhamphomyia anfractuosa*, *Rhamphomyia lineodorsata* sp. n. and *Rhamphomyia robustior*. All four species may be identified according to the key. We excluded females from the type series because of problems with exact identification of females in this group of species and because we had no pairs taken in copula. But we believe we identified them properly. Female differs from *Rhamphomyia robustior* and *Rhamphomyia anfractuosa* in lustrous abdominal tergites 6 and 7 (microtrichose in both *Rhamphomyia anfractuosa* and *Rhamphomyia robustior*), from *Rhamphomyia lineodorsata* by brown palpus (yellowish red in *Rhamphomyia lineodorsata*) and from *Rhamphomyia anfractuosa* also by front femur with two rows of ventral setae longer than depth of femur, hind tibia without broad pennate ciliation and light brownish wing (in *Rhamphomyia anfractuosa*, front femur has almost no ventral setae, hind tibia is broadly pennate both dorsally and ventrally and wing is deep brown).

#### Distribution.

Russia (Far East).

#### Dates of occurrence.

August-September.

### Rhamphomyia (Pararhamphomyia) shatalkini sp. n.

Taxon classificationAnimaliaDipteraEmpididae

http://zoobank.org/11ADE0FE-A4E1-48FB-BAF6-1923EBE2BC2A

Figs 7 , 12 , 16

#### Type material.

Holotype male: Russia, Amurskaja oblast, g. (= city) Zeja, 22.vi.1978, leg. A. Shatalkin, deposited in ZMMU; Paratypes: 2 males, same data as holotype; 2 males, same locality, 23.vi.1978; 2 males, same locality, 24.vi.1978; 1 male, same locality, 21.vi.1979 – all A. Shatalkin; 1 male, same locality, 25.vi.1982, A. Ozerov; 3 males, Russia, Juzhnoje Primorije, Kamenushka, 9.vi.1984, A. Shatalkin; 1 male, Russia, Irkutskaja o. (= oblast, = region), Listvjanka, 21.vi.1965, O.P. Negrobov; paratypes depositories: ZMMU, CULSP.

#### Diagnosis.

Species of the subgenus *Pararhamphomyia* with uniserial dorsocentrals, lustrous abdomen, tergite 6 with two thumb-like dorsomarginal processes, mesoscutum without lustrous stripes and phallus with four outgoing loops.

#### Etymology.

The species is named after Anatole Shatalkin, dipterist from Moscow Museum and collector of part of type series.

#### Description.

**Male.** Head holoptic, facets in dorsal third of eye enlarged. Frons blackish brown, light grey microtrichose, without setae. Ocellar setae fine, half as long as frons, black, ocellar triangle without additional setae. Face blackish brown, light grey microtrichose dorsally and broadly lustrous along ventral margin, 0.25 mm broad ventrally and 0.30 mm long, without setae. Occiput blackish brown, grey microtrichose, sparsely black setose, setae rather thick and short, ventrally longer and finer. Antenna black, scape brown, pedicel and extreme base of postpedicel brownish-yellow, length of antennal segments = 13: 10: 40: 17, setae on basal two segments nearly 0.12 mm long. Labrum brown, lustrous, about as long as or slightly longer than height of head. Palpus brown, short, with several setae almost 0.30 mm long. Gena narrow and lustrous, clypeus lustrous.

Thorax brownish-black, light grey microtrichose (with slight brownish-yellow tinge), scutum with somewhat darker stripes below rows of acrostichals and dorsocentrals. All setae black. Chaetotaxy: proepisternum with 10–15 setae, both propleural depression and prosternum bare; 14–20 irregularly biserial, fine acrostichals about 0.20 mm long; almost regularly uniserial slightly longer dorsocentrals (about 0.25 mm in middle of rows), ending in 2–3 strong and long prescutellars, 1 small intrahumeral, 1 strong posthumeral; postpronotum with 1 strong seta and about 15 additional finer setae; 4 notopleurals (1–2 long setae on anterior part of notopleuron); 1 supraalar and 1 equally strong prealar; 1 long and 1 shorter postalar; 3 pairs of scutellars (rarely two pairs); laterotergite with black setae.

Legs brown, lustrous, black setose. Coxae blackish-brown, microtrichose (only hind coxa with two lustrous spots anteriorly near base and at apex), black setose. One long seta in posteroapical comb of hind tibia. Fore femur with fine anteroventral setae 1/3 as long as depth of femur, posteroventral and dorsal setae shorter. Fore tibia with 4–5 strong posterodorsal setae 2× longer than width of tibia, ventral and anterodorsal setae short. Mid femur with two rows of spiny setae ventrally, anteroventrals half as long as depth of femur and densely arranged, posteroventrals sparse and longer than depth of femur, other setation short. Mid tibia with two rows of setae dorsally nearly 2× longer than width of tibia (each row consists of 4–5 setae), row of short posteroventral setae, anteroventrals more irregularly arranged and somewhat longer than posteroventrals. Hind femur (Fig. 16) with anteroventral row of rather fine setae nearly as long as depth of femur (1 or 2 of them may be stronger than remaining), other setae including posteroventrals short and fine. Hind tibia about as thick as hind femur, with 6–8 pairs of antero- and posterodorsal setae 1.5× longer than width of tibia, ventral setae short. Basal tarsomeres of fore and mid legs thin and short setose, with several short ventral spines. Basal tarsomere of hind leg thin, with several setae dorsally 2× longer than width of tarsomere and with several ventral spines slightly longer than width of tarsomere.

Wing light brownish, stigma slightly darker, veins brown, anal vein almost complete. Costal seta strong and long (several other setae between costal seta and wing base relatively strong and long), axillary angle acute. Measurements M_2_/d = 1.3–1.4, CuA_1_ ratio = 2.2–2.5, lw/ww = 2.6–3.0. Halter yellow, calypter brownish-yellow with dark fringes. Abdomen brown, lustrous (only segment 1and small spots on 3 pregenital segments microtrichose). All setae dark. Hind marginal setae on sides of tergite 2 nearly as long as segment, on segments 3–5 gradually shorter (discal setae shorter), marginals on tergite 6 short, tergite 7 bare. Dorsum of abdomen with short setae. Abdominal tergite 6 with two thumb-like processes dorsally (Fig. 7). Phallus (Fig. 12) with four outgoing loops in a single plain; phallus with hair-like “ciliation” ventrobasally and produced basal bulge; hypandrium membranose on posterior part (this part covers whole ventrobasal “ciliation” of phallus).

**Length**: Body 5.3–6.4 mm, wing 5.7–6.4 mm.

**Female.** Unknown.

#### Remarks.

Rhamphomyia (Pararhamphomyia) shatalkini sp. n. may be easily distinguished from all other Palaearctic species of *Rhamphomyia* (except unnamed species *Rhamphomyia* sp. 1) by peculiar shape of phallus forming four outgoing loops in a single plain and simultaneously tergite 6 bearing two long thumb-like dorsomarginal processes. However, the mesoscutum in the new species is entirely microtrichose but with three lustrous stripes below lines of setae in *Rhamphomyia* sp. 1. Other species with similarly formed phallus are *Rhamphomyia multisinuosa* Frey and *Rhamphomyia spectabilis* Frey, both without long thumb-like processes on tergite 6. Female of *Rhamphomyia shatalkini* remains unknown with certainty. We have at our disposal several females which may belong to either *Rhamphomyia shatalkini*, *Rhamphomyia spectabilis* or *Rhamphomyia biflexata*, but we are unable to associate them with particular males. Males of all three species have very similar microtrichosity pattern of mesoscutum which otherwise helps to associate males with females even if not taken in copula. These females differ from all other Palaearctic *Pararhamphomyia* by the following combination of characters: body entirely dark setose, dorsocentrals almost regularly uniserial, halter yellow, clypeus lustrous, both mid and hind femora broadly pennate, tibiae without pennation and abdomen lustrous except the first segment.

#### Distribution.

Russia (Far East).

#### Dates of occurrence.

June.

### Unnamed species

#### Rhamphomyia (Pararhamphomyia)

Taxon classificationAnimaliaDipteraEmpididae

sp. 1

##### Material examined.

1 male, Russia, Amurskaja oblast, g. (= city) Zeja, 26.vi.1982, M. Krivosheina, deposited in ZMMU.

##### Remarks.

Species very similar to *Rhamphomyia shatalkini* sp. n. with the exception of characters given in the key. We hesitate to describe new species from only a single specimen.

#### Rhamphomyia (Pararhamphomyia)

Taxon classificationAnimaliaDipteraEmpididae

sp. 2

##### Material examined.

1 male, Russia, Amurskaja oblast, g. (= city) Zeja, 26.vii.1978, A. Shatalkin, deposited in ZMMU.

##### Remarks.

Species very similar to *Rhamphomyia spectabilis* Frey with the exception of characters given in the key. We hesitate to describe new species from only a single specimen; moreover, we do not have *Rhamphomyia spectabilis* at our disposal.

### Key to males of Palaearctic species of the *Rhamphomyia anfractuosa* group

**Table:** 

1	Phallus forms loops in space. Abdomen microtrichose. Hind femur with ventral microtrichosity. No modifications of tergites 6 and 7. No lustrous stripes below rows of acrostichals and dorsocentrals	**2**
–	Phallus forms loops in flat plain. Abdomen lustrous. Hind femur without ventral microtrichosity. Tergites 6 and/or 7 modified. Lustrous stripes below rows of acrostichals and dorsocentrals present or absent	**5**
2 (1)	Mesoscutum dark brownish black. Abdomen dark brown. Usually three (rarely four) pairs of scutellar setae. Axillary angle acute	**3**
–	Mesoscutum light grey microtrichose. Abdomen light grey. Usually two (rarely one or three) pairs of scutellar setae. Axillary right angled	**4**
3 (2)	Mesoscutum with distinct darker stripes below acrostichals and dorsocentrals. Dorsocentrals regularly uniserial (Additional characters opposite of *Rhamphomyia nudiscutellata*: fore tibia with differentiated posterodorsal setae, wing light brown). (Additional characters: female tergites 6 and 7 lustrous and hind tibia without pennate ciliation dorsally, palpus yellowish red). Phallus as in Fig. 3	***Rhamphomyia lineodorsata* sp. n.**
–	Mesoscutum subpolished, without distinct stripes. Dorsocentrals irregularly biserial. (Additional characters: female tergites 6 and 7 microtrichose and hind tibia with broad pennate ciliation dorsally). Phallus as in Fig. 6	***Rhamphomyia robustior* Frey**
4 (2)	Hind marginal setae on tergites 5–6 nearly absent. Wings brown. Antennal stylus slightly shorter than postpedicel. Phallus with five twists (Fig. 1). (Additional characters: female front femur nearly without setae, hind tibia broadly pennate both dorsally and ventrally, wing deep brown and tergites 6 and 7 microtrichose)	***Rhamphomyia anfractuosa* Bezzi**
–	Hind marginal setae on tergites 5–6 about as long as these segments. Wings clear. Antennal stylus 1/4 as long as postpedicel. Phallus with three twists (Fig. 5). (Additional characters opposite of *Rhamphomyia lineodorsata*: fore tibia without differentiated posterodorsal setae, wing clear) (Additional characters: female fore femur with two rows of ventral setae longer than depth of femur, hind tibia without pinnate setae, wing light brownish and tergites 6 and 7 lustrous)	***Rhamphomyia nudiscutellata* sp. n.**
5 (1)	Phallus with two outgoing loops (Fig. 2)	***Rhamphomyia biflexata* sp. n.**
–	Phallus with 3 or 4 outgoing loops	**6**
6 (5)	Tergite 6 with two long thumb-like dorsomarginal processes	**7**
–	Tergite 6 without processes or with only small triangular shaped dorsomarginal projections	**8**
7 (6)	Mesoscutum microtrichose, without lustrous stripes. Dorsocentrals uniserial. Syntergosternite 8 without dorsomarginal projections. Phallus as in Fig. 7	***Rhamphomyia shatalkini* sp. n.**
–	Mesoscutum with lustrous stripes below rows of acrostichals and dorsocentrals. Dorsocentrals irregularly biserial. Syntergosternite 8 with two dorsomarginal triangular projections	***Rhamphomyia* sp. 1**
8 (6)	Tergite 6 with two small triangular shaped dorsomarginal projections. Mesoscutum with lustrous stripes below rows of acrostichals and dorsocentrals. Phallus as in Fig. 4	***Rhamphomyia multisinuosa* Frey**
–	Tergite 6 without projections. Mesoscutum microtrichose, without lustrous stripes	**9**
9 (8)	Dorsocentrals irregularly biserial. Phallus with four outgoing loops	***Rhamphomyia spectabilis* Frey**
–	Dorsocentrals uniserial. Phallus with three outgoing loops (Fig. 8)	***Rhamphomyia* sp. 2**

## Supplementary Material

XML Treatment for Rhamphomyia (Pararhamphomyia) biflexata

XML Treatment for Rhamphomyia (Pararhamphomyia) lineodorsata

XML Treatment for Rhamphomyia (Pararhamphomyia) nudiscutellata

XML Treatment for Rhamphomyia (Pararhamphomyia) shatalkini

XML Treatment for Rhamphomyia (Pararhamphomyia)

XML Treatment for Rhamphomyia (Pararhamphomyia)

## References

[B1] Barták M (1982) The Czechoslovak species of *Rhamphomyia* (Diptera, Empididae), with description of a new species from Central Europe. Acta Universitatis Carolinae - Biologica 1980(1982): 381–461.

[B2] Barták M (2002) Nearctic species of Rhamphomyia subgenus Megacyttarus (Diptera: Empididae). Acta Universitatis Carolinae Biologica 46(1-2): 3–215.

[B3] Barták M (2003) Revision of Palaearctic species of Rhamphomyia (Megacyttarus) argentea group (Diptera: Empididae). Acta Universitatis Carolinae Biologica 47: 197–245.

[B4] Barták M (2007) Five new European species of the Rhamphomyia (s.str.) albosegmentata group (Diptera: Empididae). Revue Suisse de Zoologie 114(2): 417–435.

[B5] Barták M Çiftçi MC Hasbenli A (2007) A new species of *Rhamphomyia* (s. str.) Meigen (Diptera, Empididae) from southern Anatolia. Entomological News 118(2): 143–147. doi: 10.3157/0013-872X(2007)118[143:ANSORS]2.0.CO;2

[B6] Barták M Kubík Š (2008a) New peculiar Eastern Palaearctic *Rhamphomyia* (Diptera: Empididae). Entomological News 119(4): 338–344. doi: 10.3157/0013-872X-119.4.338

[B7] Barták M Kubík Š (2008b) A new species of Rhamphomyia (Pararhamphomyia) (Diptera: Empididae) from Thailand. Oriental Insects 42: 285–289. doi: 10.1080/00305316.2008.10417552

[B8] Barták M Kubík Š (2008c) : Four new West Palaearctic species of the *Rhamphomyia* (s.str.) (Diptera: Empididae). Revue Suisse Zool. 115(1): 25–36.

[B9] Barták M Kubík Š (2009) Two new East Palaearctic Rhamphomyia (Pararhamphomyia) (Diptera: Empididae). Entomological News 120(1): 76–86. doi: 10.3157/021.120.0114

[B10] Barták M Kubík Š (2010) Three new European species of the Rhamphomyia (s.str.) melania group (Diptera: Empididae). Revue Suisse se Zoologie 117(1): 89–100.

[B11] Barták M Kubík Š (2012) A review of the Palaearctic species of Rhamphomyia subgenus Holoclera (Diptera: Empididae) with description of 5 new species. Revue Suisse de Zoologie, roč. 119(3): 385–407.

[B12] Barták M Kubík Š Civelek H Dursun O (2014) New species of *Rhamphomyia* (Diptera: Empididae) from Turkey with a key to species of the Middle East and adjacent territories. Zootaxa 3815(1): 68–78. doi: 10.11646/zootaxa.3815.1.4 24943600 10.11646/zootaxa.3815.1.4

[B13] Barták M Sinclair B (2003) Case 3269. Rhamphomyia (Rhamphomyia) Meigen, 1822 and Rhamphomyia (Pararhamphomyia) Frey, 1922 (Insecta: Diptera): proposed conservation of usage of the subgeneric names by designation of *Empis sulcata* Meigen, 1804 as the type species of *Rhamphomyia*. Bulletin of Zoological Nomenclature 60(3): 203–205. [See Opinion 2117, Bulletin of Zoological Nomenclature 62(2): 114–115]

[B14] Merz B Haenni J-P (2000) Morphology and terminology of adult Diptera. In: Papp L Darvas B (Eds) Contributions to a Manual of Palaearctic Diptera. Volume 1 Science Herald, Budapest, Hungary, 21–51.

[B15] Saigusa T (2012) A new Asio–Nearctic subgenus of *Rhamphomyia* (Diptera: Empididae: Empidinae). The Canadian Entomologist 144(2): 291–322. doi: 10.4039/tce.2012.28

[B16] Sinclair BJ (2000) Morphology and terminology of Diptera male terminalia. In: Papp L Darvas B (Eds) Contributions to a Manual of Palaearctic Diptera. Volume 1 Science Herald, Budapest, Hungary, 53–84.

[B17] Sinclair BJ Cumming JM (2006) The morphology, higher-level phylogeny and classification of the Empidoidea (Diptera). Zootaxa 1180: 1–172.

[B18] Yang D Zhang K Yao G Zhang J (2007) World Catalog of Empididae (Insecta: Diptera). China Agricultural University Press, Beijing, 599 pp.

